# Probiotics Mechanism of Action on Immune Cells and Beneficial Effects on Human Health

**DOI:** 10.3390/cells12010184

**Published:** 2023-01-02

**Authors:** Chiara Mazziotta, Mauro Tognon, Fernanda Martini, Elena Torreggiani, John Charles Rotondo

**Affiliations:** 1 Department of Medical Sciences, University of Ferrara, 44121 Ferrara, Italy; 2 Center for Studies on Gender Medicine, Department of Medical Sciences, University of Ferrara, 64/b, Fossato di Mortara Street, 44121 Ferrara, Italy; 3 Laboratory for Technologies of Advanced Therapies (LTTA), University of Ferrara, 44121 Ferrara, Italy

**Keywords:** immune cells, probiotics, human health, microbiota, beneficial microbes, microbiome, microbial modulation effects, immune system, immune modulation, immune response

## Abstract

Immune cells and commensal microbes in the human intestine constantly communicate with and react to each other in a stable environment in order to maintain healthy immune activities. Immune system-microbiota cross-talk relies on a complex network of pathways that sustain the balance between immune tolerance and immunogenicity. Probiotic bacteria can interact and stimulate intestinal immune cells and commensal microflora to modulate specific immune functions and immune homeostasis. Growing evidence shows that probiotic bacteria present important health-promoting and immunomodulatory properties. Thus, the use of probiotics might represent a promising approach for improving immune system activities. So far, few studies have been reported on the beneficial immune modulatory effect of probiotics. However, many others, which are mainly focused on their metabolic/nutritional properties, have been published. Therefore, the mechanisms behind the interaction between host immune cells and probiotics have only been partially described. The present review aims to collect and summarize the most recent scientific results and the resulting implications of how probiotic bacteria and immune cells interact to improve immune functions. Hence, a description of the currently known immunomodulatory mechanisms of probiotic bacteria in improving the host immune system is provided.

## 1. Introduction

The intestine is a complex and dynamic ecosystem which has evolved specific immune cellular characteristics over time as a consequence of incessant exposure to numerous antigens and pathogenic agents [1]. Various classes of intestinal immune cells play an important role in the host immune functions to counteract infections, and regulate the immune tolerance to commensal bacteria and ingested antigens. However, immune cell functions and activities can be modulated to a large degree by the intestinal commensal microbes with health-promoting and beneficial immunomodulatory properties [2]. Indeed, immune cells, commensal microorganisms and nutrients constantly interact with and react to one another in a stable environment in order to maintain immune homeostasis and modulate both innate and adaptive immune responses [3]. This interaction improves the functions of the immune system.

Given its particular anatomical, cellular, and molecular characteristics, the intestine is considered to be a key environment for beneficial bacteria to preserve their health-promoting effects. All mammals, including humans, spend their lives in contact with a large and varied population of different microorganisms which reside in their intestine [4]. Gut microflora comprises bacteria that have been evolutionarily selected on the basis of their capacity to survive and proliferate in the intestinal environment. One of the benefits of this interaction is that host organisms can improve their immune system by enhancing immunological responses to diseases [5], including infectious and inflammatory diseases [6]. Consistently, distinct alterations in the intestinal microbial populations can either favor or hamper alterations to the host immune functions and the related development of autoimmune diseases.

According to the Food and Agriculture Organization/World Health Organization guidelines, probiotics are ’Live microorganisms which, when administered in adequate amounts, confer health benefits on the host’ [7,8]. Nowadays, probiotics represent an important group of beneficial consumed/supplemental microorganisms that can live in foods/supplements and in the intestine [9]. When consumed, probiotics can positively influence the composition of intestinal microflora and interact with different immune cells, thus improving immune functions [10,11,12,13,14]. It is therefore widely acknowledged that probiotics present health-promoting and immunomodulatory properties [8,15]. Indeed, these microorganisms are highly reliable in preventing the onset of various disorders [16,17]. As a consequence, consumed probiotics might provide cost-effective alternative solutions for disease management [18,19,20,21]. Although the beneficial properties of probiotics are well known, there is a need to understand the mechanisms underlying their interaction with immune cells in stimulating immunomodulatory effects [22]. Moreover, the identification of novel and emerging probiotic strains with similar properties is also necessary [9,23,24,25].

Notably, the majority of currently available studies are focused on the metabolic properties of probiotics, while there is still relatively little research on their immunomodulatory effects. The mechanisms behind the interaction between host immune cells and probiotics have only been partially described in the literature.

The present review aims to collect and describe the main scientific results published to date and their implications on how immune cells and probiotics interact to enhance the immune function. A description of current knowledge on the immunomodulatory properties of probiotic bacteria in improving the host immune system is therefore provided.

## 2. The Immune System of the Gut

**Figure 1:**
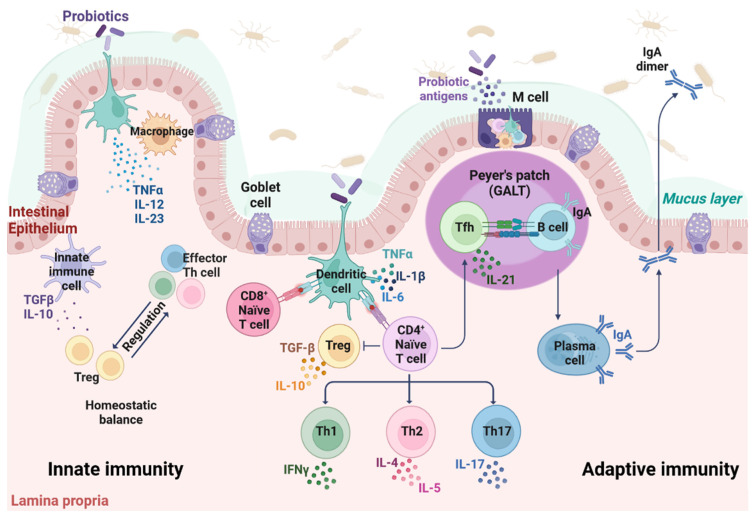
Schematic representation of the interaction between host intestinal immune cells and probiotics. Probiotics play a role in host innate and adaptive immune responses by modulating immune cells such as dendritic cells (DCs), macrophages, and B and T lymphocytes. Interactions between host intestinal cells and probiotics mainly occur at the surface of the intestinal barrier, including the intestinal epithelium and the underlying lamina propria. Intestinal microbiota is separated from the intestinal epithelium by a mucus layer secreted by goblet cells. Consumed probiotic bacteria adhere to intestinal epithelial cells and activate them by pattern recognition receptors (PRRs). Cytokines stimulated by probiotic bacteria lead to the activation of T regulatory (Treg) cells, which maintain immune homeostasis in the intestinal mucosa. Tregs are effective suppressors of the immune response and play a key role in limiting immune response. Intestinal antigens are transferred to DCs via specialized enterocytes known as microfold cells (M cells), which are located in the epithelium overlying Peyer’s patch. Probiotics are processed directly by DCs in lamina propria in the intestinal lumen. Intestinal DCs can activate CD8+/CD4+ naïve T cells and direct helper T cell responses towards Th1, Th2, Th17, or regulatory patterns. The Th1 immune response is mainly characterized by interferon (IFN)-γ production and is involved in cell-mediated immunity. The Th2 immune response includes interleukin (IL)-4, IL-5 release, thus inducing humoral immunity. The Th17 immune response is characterized by IL-17 production. Induction of Tregs releases IL-10 or transforming growth factor (TGF)-β. In addition, probiotics induce maturation of B cells into immunoglobulin (Ig)A-producing plasma cells. Intestinal epithelial cells release cytokines and chemokines, creating a microenvironment in the lamina propria of the intestine that allows the clonal expansion of B cells to produce IgAs. IgAs migrate through the epithelium into the mucus layer where they control bacterial adhesion to the host tissue.

The gut immune system provides physical barriers, that is the epithelium and underlying connective tissue, namely lamina propria, which contains the immune effector cells [26]. The lymphoid tissue associated with the intestinal tract is the gut-associated lymphoid tissue (GALT), which also presents important immune functions. GALT belongs to the mucosa-associated lymphoid tissue (MALT) and makes up the most extensive part of the total immune capacity (Figure 1). It represents a massive source of T and B cells that migrate to effector sites to induce immune responses [27]. Different dendritic cell (DC) populations are also found in GALT [28]. Moreover, GALT comprises Peyer’s patches, which are follicle-associated epithelia localized throughout the intestinal epithelium and in secretory sites within the mucosa. Peyer’s patches play an important immunological role in monitoring intestinal bacteria and therefore preventing intestinal pathogenic infections. Given the anatomical structure and tissue composition of the intestine, the epithelial layer can be considered as a front line for external stimuli, while GALT mediates adaptive immune responses [28]. DCs capture antigens from epithelium and microfold (M) cells, in order to activate T cells by antigen recognition [29].

Given its cytological composition and its histological architecture, the intestine is considered the largest immunological organ as it contains approximately 70–80% of all IgA-producing B cells [30]. IgAs are proteolytic-resistant antibodies locally synthesized in effector tissues which, in turn, are particularly important in the mucosal membrane immune function [31,32]. The mucosal immunological function of IgAs is to provide protection for mucosal surfaces, by binding to and neutralizing foreign antigens from pathogenic agents/toxins. IgAs therefore inhibit microorganism adhesion to intestinal epithelial cells and subsequent penetration. However, recent evidence suggests that IgAs are emerging as inflammation players, both at mucosal and non-mucosal sites [33].

Two distinct immune responses, i.e., innate and adaptive immunities, synergistically work together in the gut to protect organisms against pathogens. In particular, antibody-mediated (or humoral) and cell-mediated immunities represent two types of adaptive immune responses [34,35,36,37]. The innate immunity is not specific as it provides the first unspecific line of defense against offensive, external targets/agents. Key players in innate immunity, which are generally related to inflammation, include physical/chemical barriers, such as skin and mucous membranes, immune cells such as DCs, monocytes, macrophages, neutrophils and natural killer (NK) cells, as well as molecules such as cytokines. Adaptive/acquired immunity can be viewed as a second, extremely specific line of defense against offensive targets. Two lymphocyte types, i.e., B and T cells, carry out the adaptive immunity through different modalities, including antibody responses, by producing immunoglobulins (Igs) [38], and cell-mediated immune responses [39]. In adaptive immunity, the interaction between antigen-presenting molecules, such as major histocompatibility complex (MHC) proteins, expressed on the surface of antigen-presenting cells (APCs), and T cell receptors expressed in helper (CD4) T lymphocytes (Th lymphocytes), mediates CD4+ lineage commitment, activation and homeostasis. In the cell-mediated immunity, CD8+ helper and CD4+ cytotoxic T cells express CD8 and CD4 co-receptors on their surfaces, which recognize antigen-MHC classes I and II complexes, respectively [40,41]. Moreover, MHC class II complex is expressed in DCs, which are APCs that connect innate and adaptive immunity. Naïve CD4+ T cells can differentiate into various subsets of Th cells based on specific cytokine secretion profiles through maturational processes that are induced by immune antigenic stimulation orchestrated by APCs such as DCs [42,43]. Cytokines include different classes of small proteins that act as immunomodulating agents [44,45,46]. Anti-inflammatory cytokines, such as interleukin-10 (IL-10), acting as immunoregulatory molecules, can control pro-inflammatory cytokine response. Specific pro-inflammatory cytokines involved in intestinal inflammation include interferon (IFN)-γ, IL-12, IL-1β, and tumor necrosis factor (TNF)-α [47,48,49].

The intestine is a complex multifunctional organ covered by a single layer of a viscoelastic mucus, where anti-microbial peptides/proteins, alongside a variety of antigen-specific mucosal effector cells, act synergistically to exert immune responses. This mucus barrier physically prevents the underlying epithelium and lamina propria to be reached by external dangerous factors/agents [50]. Moreover, the mucus is composed of mucins, which are large O-linked glycans-based glycoproteins secreted by Goblet cells scattered throughout the intestinal epithelium [50]. In physiological conditions, mammals, including humans, live in homeostatic symbiosis with their intestinal microbiota. The mammal host sustains its microbiota with nutrients and a stable and protective environment. Gut microbiota, in turn, provides appropriate nutritional contributions and maintains physiologically healthy gut mucosa [51,52,53]. Therefore, the intestine can be considered to be a large bioreactor containing ~10^14^ bacteria, which act as beneficial microbes, thus improving host nutrient metabolism [54,55].

Gut microflora improves digestion and the assimilation of diet nutrients as well as cell debris and other host cell components; a role in xenobiotic and drug metabolism has also been documented [56,57]. Commensal microbiota improves host nutritional needs in supporting normal health by synthetizing short chain fatty acids (SCFAs), vitamins, and even essential amino acids by degrading different polysaccharides/proteins that cannot be processed in the intestine [58,59,60,61]. Overall, the presence of commensal microbiota in the gut ensures mechanical and structural integrity as well as the barrier function of intestinal mucosal surfaces, thus protecting the intestine [6]. One of the most important functions of the intestinal commensal microbiota is the maintaining of a healthy immune system [54,62]. Indeed, numerous beneficial bacteria species colonizing the intestine play an important role in immune gut homeostasis [63,64,65,66].

An intriguing aspect of the intestinal immune system is how it can distinguish commensal bacteria from harmful pathogenic agents. So-called immunological tolerance guarantees the prevention of an immune response against commensal bacteria whose cellular components present a certain degree of similarity from pathogenic bacteria. In a similar fashion, oral tolerance is an active process comprising immune exclusion and immunosuppressive mechanisms to dietary innocuous antigens. These functions are enabled by a complex system of receptors named PRRs, including Toll-like receptors (TLRs), which are expressed on the surface of intestinal sentinel cells, such as macrophages and DCs. These receptors guard intestinal lumen and improve the immunological defense mechanism in terms of pro-/anti-inflammatory cytokine release [67]. In contrast, nucleotide oligomerization domain (NOD)-like intracellular receptors are intracellular receptors, which scan the cytoplasmic compartment [68]. All these molecules can recognize and bind to numerous microbial ligands and, in turn, allow the discrimination of commensal bacteria from harmful pathogens. The mechanisms of immunological tolerance and immunogenicity work together to maintain mucosal immune homeostasis. Intestinal epithelial cells play a role in this context by maintaining the homeostatic balance between tolerance and immunity [8]. The colonization of gut microflora is regulated by the immune system, which interferes with the ability of intestine microorganisms to bind to the mucosa. At the same time, the intestinal microflora and their metabolites can actively modulate the immune system [69,70,71,72]. Immunomodulation mechanisms mainly include (i) macrophage activation by probiotics signaling, (ii) stimulation of IgA-producing cells and neutrophils, (iii) peripheral Ig production stimulation, (iv) mucus production stimulation, and (v) pro-inflammatory cytokines release inhibition [73,74].

## 3. Probiotic Bacteria

The early identification of probiotics as natural and beneficial gastrointestinal microbiota dates back to the end of the 19th century when colonizing microorganisms in the digestive tracts of asymptomatic healthy individuals were described. It is now well accepted that probiotic bacteria are commensal microorganisms living in a large variety of foods and in the gastrointestinal tract [9,75,76]. These bacteria are able to compete with harmful microbes and colonize the intestine. Moreover, probiotics can provide health benefits when consumed, by improving or restoring physiological intestinal microflora composition/activity.

Probiotics are, largely, Gram-positive bacteria that include species belonging to the lactobacillus and bifidobacterium genera [77,78,79,80]. Specific escherichia coli [81], enterococcus [82,83], pediococcus, and yeast species [84,85], including saccharomyces boulardii, are examples of other non-pathogenic species with probiotic features [86,87,88,89]. Additional intestinal commensal bacteria, such as streptococcus oralis and salivarius, have been reported to confer beneficial effects on health [90,91,92].

Several beneficial effects of probiotics, on the intestinal homeostasis, have been reported [12], such as (i) amelioration of innate and adaptive immune responses and related anti-pathogenic/inflammatory activities [12,93], (ii) enhancement of bioavailability of certain natural or metabolic components and essential nutrients [94,95], and (iii) food intolerance decrease among susceptible subjects [96]. In other words, similar to gut commensal microbiota, consumed probiotics have been shown to positively affect the entire organism by improving digestion and immunity [97].

Given the highly different nature and chemical composition of probiotic molecular effectors, probiotics accomplish their beneficial effect through various mechanisms. Probiotics can directly exert their beneficial effects using different cross-feeding mechanisms [98,99], as well as mediating direct cell-to-cell contact in the intestine. Molecularly, probiotics secrete a huge number of diverse molecules in the intestinal milieu, which act as effectors in a complex cross-talk among gut microflora, intestinal immune, and epithelial cells. These molecular effectors mainly consist of (i) proteins of different natures, which are either localized in microbial surfaces or secreted into the extracellular compartment, (ii) low molecular weight peptides and/or amino acids, and (iii) bacterial DNA, (iv) SCFAs [100,101]. Similar to bacterial cell surface fragments, probiotic antigens can cross the intestinal barrier and stimulate the immune system [102]. These multiple classes of compounds are essential for various host physiological functions.

In contrast to classical supplements that mainly act on specific parts of the body, probiotic products can be considered to be a reliable asset for the host in ensuring benefits to multiple sites of the body [103,104]. The beneficial properties of consumed probiotics on host immune system improvement have been demonstrated in the treatment of several conditions and diseases, including allergies, diarrhea, inflammatory bowel disease (IBD), irritable bowel syndrome (IBS), infections, and infant colic, as well as certain forms of cancer. An increasing number of consumed probiotic bacterial species have been identified as improvers of antibiotic therapies by reducing adverse effects [103,105], whilst at the same time enhancing mucosal immunity [5,106]. They can confer benefits to patients under therapy with broad-spectrum antibiotics by restoring a healthy intestinal microflora [74,107]. Additional beneficial effects on health are under investigation [76,108,109,110,111].

### The Gut-Central Nervous System and Gut-Respiratory System Axes

The intestinal microbiota plays essential immunomodulatory activities, which can occur both locally and systemically. A complex biochemical signaling takes place between the gastrointestinal tract and different systems of the body, such as the central nervous and respiratory systems. The role of the gut microbiota in this interplay is defined as microbiota–gut–brain and microbiota–gut–lung axes. While the commensal bacteria carry out their functions/activities in the gut, they are able to act distally in other anatomical districts, such as brain and lungs. Modifications in the microbiome which can be induced by an altered homeostasis, specific conditions and/or dietary modifications, might alter the immune function and homeostasis in the central nervous system and respiratory tract [112,113].

The gut-brain axis relies on a complex neural and hormonal network. This network allows the formation of a bidirectional cross-talk between the gut and the brain and vice versa, which are both continuously interacting in both physiological and pathological conditions [114]. In vivo studies have demonstrated that gut microbiota is important for the development and maturation of the enteric nervous system and the hypothalamic pituitary adrenal axis [115]. Moreover, lack of intestinal commensal bacteria has been related to an impaired expression and turnover of neurotransmitters [115]. The gut microbiota also mediates the modulation of enteric sensory afferents and produces metabolites which target the nervous system cells [115]. Probiotics can therefore improve the immune function. For instance, in vivo evidence demonstrated that the administration lactobacillus helveticus R0052 and bifidobacterium longum R0175 can attenuate the hypothalamic-pituitary-adrenal axis and the autonomic nervous system activities [115]. The hippocampal neurogenesis and the expression in hypothalamic genes involved in synaptic plasticity has also been reported following probiotic administration [115]. In turn, the central nervous system can induce modifications in the (i) production of the intestinal mucus and biofilm, (ii) intestinal motility and permeability, and (iii) intestinal immune functions [115].

Despite the respiratory tract historically being considered to be sterile, broad evidence demonstrates that this anatomical district in healthy individuals harbors numerous microorganisms, which differ considerably between the upper and lower respiratory tract [112]. The predominant microbial communities in the respiratory tract comprise (i) firmicutes and actinobacteria phyla in the nasal cavity, (ii) firmicutes, proteobacteria, and bacteriodetes in the oropharynx, and (iii) bacteroidetes, and firmicutes in the lungs. A continuous cross-talk between the airway microbiota and host immune cells has been described [112,116]. In addition to the airway resident microbes and its local activity, the gut microbiome can modulate the respiratory homeostasis. This phenomenon can occur by the production of molecules such as the pathogen-associated molecular patterns (PAMPs) which are translocated from the intestine to the lungs in order to modulate the immune function of the respiratory tract [117]. Therefore, microbial metabolites present important immunomodulatory functions that can be harnessed to treat specific diseases of the respiratory tract.

## 4. Immune Modulatory Mechanisms of Probiotic Bacteria

**Figure 2:**
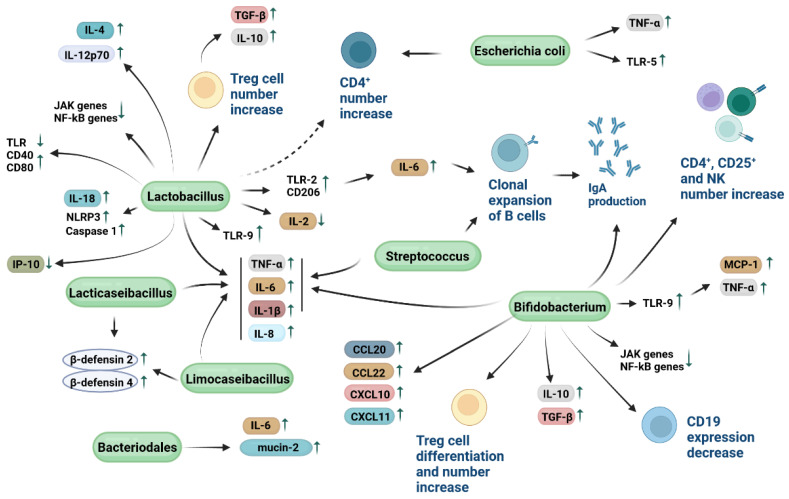
Mechanisms of action of probiotic bacteria. Lactobacillus can (i) stimulate T cell regulatory cells (Treg cells) to produce TGF-β, interleukin-10 (IL-10), and IL-8, (ii) increase levels of secreted IL-6 secreted in a Toll-like receptor (TLR)-2-dependent manner, thereby inducing the clonal expansion of all IgA-producing B cells, while also stimulating the expression of macrophage mannose receptor CD206, (iii) inhibit the expression of Janus kinase (JAK) and nuclear factor kappa-light-chain-enhancer of activated B cells (NF-κB) genes, (iv) increase the release of IL-12p70 and IL-4, (v) reduce the TLR expression and increase CD40 and CD80 expressions, (vi) degrade the proinflammatory chemokine IFN-γ-inducible protein 10 (IP-10), (vii) increase the expression of TLR-9, and (viii) favor the expression of nucleotide binding oligomeric domain-like receptor protein 3 (NLRP3), cysteine proteinase-1 (Caspase-1), and IL-18. Lacticaseibacillus and limocaseibacillus can induce β-defensins 2 and 4 and IL-8 expressions. Distinct studies reported opposing data on TLR expression. Bifidobacterium can (i) inhibit the expression JAK and NF-κB genes, (ii) favor the overexpression of IL-10 and TGF-β, while, at the same time, stimulate the production of IgAs, (iii) favor Treg cell differentiation, (iv) increase the total helper (CD4+) and activated (CD25+) T lymphocytes and NK cells, (v) reduce the expression of CD19 on B cells, (vi) induces the production of monocyte chemoattractant protein 1 (MCP-1) and TNF-α through TLR-9 stimulation, and (vii) increase the number of Foxp3(+) T regulatory cells and the release of CCL20, CCL22, CXCL10, and CXCL11. Escherichia coli can induce the expression of TLR-5 and TNF-α as well as increase the number of CD4+ cells. Bacteroidales stimulates the release of IL-6 accompanied by the expression of mucin-2 and claudin-1. Lactobacillus, lacticaseibacillus, limocaseibacillus, bifidobacterium, and streptococcus can favor the release of TNF-α, IL-6, IL-1β. Streptococcus can induce clonal expansion of B cells stimulated to release IgAs. Dash arrow: conflicting data have been reported on the effect of lactobacilli in increasing CD4+ T cell number.

Immunomodulatory activity is one of the most important function of probiotics (Figure 1 and Figure 2, Table 1) [12,118]. This activity has been demonstrated by the interactive potential of probiotic bacteria with immune cells, such as lymphocytes, monocytes, macrophages and DCs, as well as intestinal epithelial cells. Probiotics can improve the intestinal immune function by eliciting B cells to produce IgAs. The oral administration of various probiotics, such as lactobacillus casei, acidophilus, rhamnosus, delbrueckii subsp. bulgaricus, plantarum and lactis, as well as streptococcus thermophilus, has been reported to increase the number of intestinal IgA-producing cells in a dose-dependent manner. Probiotics can induce clonal expansion of B cells stimulated to release IgAs, without perturbing the CD4+ T cell count [119]. Additional studies reported that probiotic bacteria can prompt the release of secretory IgAs [12]. Upon the oral administration of probiotic bacteria such as lactobacillus casei CRL 431 and lactobacillus helveticus R389, an increase in IL-6 levels secreted in a TLR2-dependent manner has been described to be the cause of an intestinal IgA-producing cell number rise without a simultaneous CD4+ T-cell number increase. This evidence suggests that lactobacilli can elicit the B cell clonal expansion through IL-6 production in order to release IgAs (Figure 2) [120].

In summary, probiotic bacteria are able to induce the luminal section of IgAs in order to improve mucosal/systemic immunity [172]. IgAs are released in the intestinal lumen in large amounts to prevent dangerous bacteria from reaching the intestinal epithelium, thus limiting gut colonization [173]. A similar anti-microbic function relies on the ability of probiotics to change the composition of viscoelastic mucus in the mucosal barrier by influencing mucin expression [174]. Moreover, probiotics can prevent the adhesion to and proliferation of harmful pathogens on the mucosal layer, thus protecting the intestinal enterocytes and lamina propria [175]. The main adhesion mechanisms mediated by probiotics encompass both non-specific physical binding modalities as hydrophobic interactions and specific adhesion molecules located in the probiotic bacterial wall components comprising (i) mucus-binding proteins, which are surface adhesive proteins containing the mucus binding (Mub) and/or the mucin binding (MUCin-Binding Protein (MucBP)) domains, (ii) fimbriae or pili, including Type IV pili and/or minor fiber components known as sortase-mediated pilus assembly (Spa)-A, -B and -C, which are thin proteinaceous extensions from bacterial cells, and (iii) fibronectin binding proteins (FBPs) and surface layer proteins (SLPs) [175]. Consumed probiotics can stimulate beneficial commensal microflora colonization [176]. Therefore, the positive effect of probiotics on the immune functions can be brought about by changing the activity and/or composition of the intestinal immune cells and microbial community [8,177].

Orally administered probiotics are able to ameliorate host immunity in elderly people [178]. The immune function of the host has been reported to be significantly enhanced in elderly subjects introducing live bifidobacterium lactis HN019 into their diet [135]. The reported enhanced level of IFN-α upon stimulation of cultured PBMCs isolated from healthy volunteers has been hypothesized as the underlying immunomodulatory mechanism. Similarly, the leucocyte count in elderly volunteers has been reported to rise upon the consumption of milk supplemented with active bifidobacterium animalis DN-173 010; this increase leads to improved immunity [124]. The consumption of certain types of probiotics gave significant benefits in immune cells in a subclass of patients particularly prone to gastrointestinal infections [179]. For instance, CD4+ cell count has been reported to increase following the consumption of yogurt with lactobacillus rhamnosus GR-1 and reuteri RC-14 [160]. It has also been suggested that administered probiotics are helpful in attenuating inflammation in this class of patients [180].

Rotavirus is the main causative factor of gastroenteritis and diarrhea worldwide in children, while being responsible for about 20–25% of diarrhea-associated infantile deaths, especially in developing countries [181]. The efficacy of lactobacillus reuteri ATCC 55730 in improving the response to rotavirus has been evaluated and demonstrated in an early study conducted on a group of children with rotavirus diarrhea [163]. Upon administration, the probiotic increased the release of IgAs, while reducing the duration of diarrhea in a dose-dependent manner. Similarly, lactobacillus casei and rhamnosus GG proved to be effective in stimulating the immune response against rotavirus in a group of children with acute diarrhea [148,182]. Nowadays, the efficacy of probiotics in immunomodulating the response against rotavirus is well documented [183]. A large variety of probiotics are currently recommended for managing diarrhea, infantile colic, and other gastrointestinal diseases [184,185]. However, it should be underlined that lactobacilli are currently considered the most effective bacteria in the treatment of these diseases.

Evidence denotes that intestinal microbiota may influence the immune response to vaccination [186,187]. The performance of oral vaccines is notoriously poor in developing countries where vaccinated children frequently present either dysbiosis or impaired intestinal microbiota as a result of the loss of gut commensal microorganisms able to promote a proper immunity [107,188]. Conditions of intestinal dysbiosis can lead to IBD, which is a group of disorders that cause chronic inflammation, including ulcerative colitis and Crohn’s disease [189]. Children experiencing intestinal dysbiosis can therefore develop IBD-related diseases and inflammatory complications, such as allergic sensitization, as a consequence of the impairment of their intestinal immune function [190,191]. In contrast, immune response has been reported to be improved significantly in children under therapy with anti-parasitic drugs, which can improve the intestinal immunity [192]. Moreover, an animal model-based study reported a strong correlation between TLR-5 expression and the administration of a flagellated strain of escherichia coli, while a concomitant enhancement of the immune response to inactivated influenza vaccine was found [138]. In particular, TLR5 deficiency or antibiotic treatment has been reported to have no influence on alum-adjuvanted or live-attenuated vaccines. At the same time, unadjuvanted vaccines proved to prompt antibody responses through TLR5-probiotic dependent mechanisms [138]. Probiotic immunomodulatory function via TLR activation has been described in additional studies [193,194]. These studies cumulatively underlined the beneficial effect of probiotic bacteria in improving vaccine efficacy.

Although the molecular mechanisms and factors mediating the probiotic-immune cell interaction processes have to a certain extent been identified, several surface and cell-envelope proteins and molecules of probiotic origin have been reported to play important roles in this interaction [98]. Similarly to antigenic molecules, it has been demonstrated that probiotic particles can remain stable until 72 h inside the immune cells [147]. Probiotics can therefore induce the expression increase of TLR-2 and mannose CD206 receptors on the surface of both DCs and macrophages, leading to the stimulation of an adaptive immune response [145]. A significant number of studies identified both bacterial proteins and other non-proteinaceous molecules, namely teichoic acids (TA) comprising lipoteichoic acids (LTA), exopolysaccharides (EPS), and peptidoglycan (PG), as bacterial molecular effectors that mediate immunomodulatory mechanisms.

Regarding probiotic proteins, SLPs have been reported to be involved in the interaction between lactobacilli and DC cells. In particular, SLPs strongly favor the binding between lactobacillus acidophilus NCFM and a specific CD receptor, named DC-SIGN. This interaction stimulates the release cytokines such as IL-12p70, TNFα, and IL-1β, while T cells primed with lactobacillus acidophilus NCFM stimulated DCs to release the IL-4 cytokine [142]. Additional evidence indicates that proteins located in the PG layer, such as flagella, pili and fimbria can be specifically recognized by host immune cells. Among them, Spa-CBA pilus fibers in the probiotic lactobacillus rhamnosus GG have been well characterized for their adhesion properties with host cells [195]. Soluble proteins that are produced by probiotic bacteria present different roles in the microbe-immune cell interplay. In lactobacillus plantarum, the secretion of members belonging to a family of serine-threonine rich proteins, namely STp, can favor bacterial aggregation [98], while at the same time STp can modulate the DC phenotype of patients affected by ulcerative colitis; conditioning of DC with STp can reduce TLR expression and increase CD40 and CD80 expression [157]. It has also been demonstrated that the immunomodulatory effect of bifidobacterium longum is partially mediated by the secretion of the serine protease inhibitor serpin, which binds to and inactivates the human neutrophil and pancreatic elastase [136]. Moreover, in lactobacillus paracasei, the secretion of protease lactocepin has been reported to contribute to the host intestinal homeostasis by exhibiting anti-inflammatory effects. In particular, lactocepin is able to selectively degrade the proinflammatory chemokine IFN-γ-inducible protein 10 (IP-10) which play a role in lymphocyte recruitment [155].

Among non-proteinaceous probiotic molecules, a growing number of studies reported data on LTAs and EPS as immunomodulatory molecular effectors. Indeed, anti-inflammatory properties of TAs from lactobacillus plantarum have been reported in terms of production profiles of monocytes and PBMCs exposed to these molecules [98]. LTAs belong to a larger class of linear polymers bonded either to PG (wall TAs) or to the cytoplasmic membrane (membrane TAs) of probiotic bacteria. Moreover, an animal colitis model-based study indicated that the administration of lactobacillus plantarum LTA resulted in an improved disease outcome [158]. The immunomodulatory capacity of lactobacillus plantarum LTA was also found to be TLR-2-dependent. These findings indicate that LTAs can modulate the immune responses of the host. EPS from lactobacillus and bifidobacterium strains have been reported to play a role in preventing pathogen invasion. EPS showing immunomodulatory properties have been identified in (i) lactobacillus plantarum N14 strain, as being reported to molecularly interact with the TLR family protein RP105/MD1 complex [159], (ii) bifidobacterium breve UCC2003 and bifidobacterium animalis subsp. lactis, by facilitating commensal-host interaction through immune modulation and pathogen protection [98,128,129]. Lastly, epigenetics mechanisms [196], such as differential methylation of probiotic’s DNA, play an immunomodulatory role in the host. Indeed, unmethylated CpG motifs in bifidobacterium DNA can induce the production of monocyte chemoattractant protein 1 (MCP-1) and TNF-α through TLR-9 stimulation on macrophages surfaces, thus leading to a Th1 orientation of the immune system [123].

As mentioned before, pattern recognition receptors such as TLRs are expressed on the surfaces of host cells and can be recognized by probiotics [12]. This interaction ultimately leads to the regulation of crucial immunoregulatory signaling pathways, thus favoring the release of NF-kB and mitogen-activated protein kinases [178]. An early study demonstrated that lactobacillus casei CRL 431 and lactobacillus paracasei CNCM I-1518, were able to bind to intestinal epithelial cells by interacting with TLRs, leading to the initiation of the immune stimulation processes represented by IL-6 release and macrophage chemoattractant protein 1 expression. Stimulated intestinal epithelial cells can, in turn, stimulate immune cells located within the intestinal lamina propria, favor the cytokine release by T cells, and activate innate immune responses [145].

Probiotics such as lactobacillus helveticus IMAU70129, lactobacillus rhamnosus GG, lactobacillus rhamnosus KLSD, and lactobacillus casei IMAU60214 can stimulate the innate immunity by increasing the phagocytic and bactericidal activities of human monocyte-derived macrophages and the levels of reactive oxygen species (ROS) in vitro, as well as increase the nuclear translocation NF-κB pp65 and TLR2-dependent signaling [150]. Similarly, an additional in vitro study reported that the phagocytosis of macrophage cells as well as the expression of IL-1β and CD80 have been reported to increase the pre-treatment with lactobacillus johnsonii NBRC 13952 [152].

In summary, various probiotic molecules have been reported to mediate the probiotic-immune cell interaction. It should be underlined, however, that in spite of the fact that some promising results have been obtained, further research is needed to elucidate the mechanisms of interaction between probiotic components and host immune cells.

## 5. Host Cytokine Release and Probiotics

The specific interaction among intestinal immune, epithelial cells, and probiotic bacteria can promote a signaling cascade in terms of pro- and anti-inflammatory cytokines release, which can modulate the immune function [147,197].

Evidence indicates that distinct classes of probiotic bacteria are capable of modulating the inflammatory response by acting as immunoregulatory effectors. Probiotics can therefore stimulate an innate, non-specific, immune response in which innate immune cells locally detect infection or tissue injury [65,73,198,199,200]. Probiotics can either directly or indirectly influence the immune response by stimulating the production of cytokines, including ILs, IFNs, TGFs, TNFs, and chemokines by either immune cells comprising DCs, lymphocytes, macrophages, mast cells, granulocytes, or intestinal epithelial cells [130,201,202]. For instance, while probiotics with immunoregulatory characteristics can induce the release of IL-10 and Treg cells, immunostimulatory probiotic bacteria have been demonstrated to allow IL-12 production, which, in turn, develops Th1 cells as well as activates NK cells [73]. In the first case, probiotics with an immunoregulatory function can be employed to manage autoimmune diseases [203], allergy, IBD as well as inflammation [204]. In the second case, immunostimulatory probiotics can boost responses against infections and/or cancer cells.

Probiotics such as lactobacillus casei CRL 431 have been demonstrated to maintain the intestinal homeostasis by stimulating the release of IL-10 by Th2 lymphocytes and macrophages [146]. Moreover, an early study conducted in vitro with colorectal adenocarcinoma cells/peripheral blood mononuclear cells (PBMCs) co-cultures reported that the production of pro-inflammatory cytokines IL-1β, IL-8, and TNF-α can be induced by lactobacillus sakei [151]. The authors concluded that the sensitization of colorectal adenocarcinoma cells by neighboring immunocompetent cells constitutes a crucial step for the recognition of non-pathogenic bacteria. Since the two cell types were not co-cultured by direct cell-to-cell contact, the involvement of surface molecules, such as the recognition of non-classical restriction elements, was excluded. The mechanism was therefore mediated by a soluble factor, such as proinflammatory molecules [151]. The same study also reported that the probiotic lactobacillus johnsonii can stimulate the production of anti-inflammatory cytokine TGF-β. A significant decrease in pro-inflammatory cytokine TNF-α expression and an increase in CD4+ cell number has been reported in co-cultures of intestinal mucosa from a Crohn’s disease patients with lactobacillus casei, bulgaricus, and crispatus, and even escherichia coli [139,205]. Therefore, different probiotic strains can de facto interact with immunocompetent cells on the intestinal mucosal surfaces through the local modulation of proinflammatory cytokines [139]. An early study on lactobacillus casei suggested that the probiotic might be able to enhance the intestinal immune system by increasing specific markers of innate immune response cells such as CD-206 and TLR-2, with no modification in the number of T cells [147]. More recently, Reséndiz-Albor et al. reported that epithelial cells treated with various lactobacillus and bifidobacterium strains overexpressed IL-6, IL-10, and TGF-β, while at the same time stimulating the production of IgAs [206]. Hence, probiotics are able to simulate the Ig receptors of intestinal epithelial cells. Co-culture systems of human colon cells and colon cancer cells with PBMCs stimulated with streptococcus thermophilus, lactobacillus rhamnosus, casei, acidophilus, and bifidobacterium bifidum and longum, resulted in an increased production of TNF-α and IL-1β accompanied by a reduced production of various cytokines [130]. To determine the immunological mechanisms that underpin tolerance to bowel commensals, an additional study evaluated the cytokine responses of DCs and T cells after exposure to lactobacillus reuteri 100-23. Results indicated an increased production of anti-inflammatory cytokines IL-10 and TGF-β, in parallel with a reduction of IL-2. Probiotics can therefore stimulate the development of an increased number of Treg cells [165].

A recent meta-analysis conducted on a total of eight studies assessing probiotic intakes and salivary cytokines and Igs suggested that local administrations of probiotics such as lactobacillus casei Shirota, fermentum and rhamnosus, as well as bifidobacterium animalis, might influence the release of some salivary cytokines [125]. Evidence demonstrates that lacticaseibacillus paracasei SD1, rhamnosus SD4 and SD11, and limosilactobacillus fermentum SD7 can induce human β-defensin 2 and 4, IL-1β, IL-6, IL-8, and TNF-α expressions in human gingival epithelial cells [166,207]. An additional recent study conducted on a model of human colorectal adenocarcinoma cell line indicated that sonicated probiotics lactobacillus spp. and bifidobacterium spp. are able to induce the downregulation of JAK genes and TIRAP, IRAK4, NEMO, and RIP genes in the NF-kB pathway [208]. Moreover, IL-6 production has been reported to decrease after probiotic stimulation in the Aghamohammad et al. study [208], while opposing data have also been reported on IL-6 release [131]. The use of different experimental models can be considered to be an explanation for this discrepancy.

These data support the view that the nutritional supplementation of probiotics can reduce intestinal inflammation-associated diseases, such as IBD, as well as modulate gene expression in human cells [208]. Moreover, lactobacillus rhamnosus GG has been reported to interact with macrophages and further improve their ability to discriminate between pathogenic bacteria and probiotics by an INF-mediated TLR gene regulation mechanism [98,209].

In order to alleviate inflammation, probiotics such as bacteroidales can favor the production of IL-6 and promote the secretion of mucin-2 and claudin-1 [210]. Therefore, since IL-6 is necessary for the clonal expansion of B cells, probiotics can positively modulate B cell activity [210]. Similarly, previous in vitro data describe both bifidobacterium breve IPLA 20004 and bifidum LMG13195 being able to improve the intestinal barrier function by eliciting chemokine production [132]. Co-cultures of these probiotics with a human colorectal adenocarcinoma cell line favored Treg cell differentiation and the release of CCL20, CCL22, CXCL10, and CXCL11 capable of recruiting effector immunoreactive lymphocytes [132]. In particular, CCL20 (or macrophage inflammatory protein-3α (MIP-3α)), can attract CCR6-expressing lymphocytes and DCs to the mucosal surfaces, organizing lymphoid tissues, such as Peyer’s patches, mesenteric lymph nodes, and GALT. Thus, upon probiotic stimulation, intestinal epithelial cells can potentially connect both innate and acquired mucosal immunities by upregulating CCL20 [132].

These studies cumulatively indicate that the release of various classes of both cytokines and chemokines can be favored following probiotic administration [211]. However, the molecular mechanisms at the basis of these immunological processes remain to be determined.

## 6. Animal Model-Based Studies with Probiotics

Experimental data obtained in vivo reported that specific probiotic bacteria may provide benefits in host immune homeostasis and immune function, while being reliable in preventing or treating diseases [97,212,213,214,215,216,217]. An early in vivo study reported that oral administration of lactobacillus gasseri SBT2055 (LG2055) induced IgA production and increased the rate of IgA^+^ cell population in Peyer’s patch and in the lamina propria of the mouse small intestine. Mechanistically, this immunomodulatory effect might result from the stimulation of TGF-β expression and activation of TLR-2 signalling pathways [154]. Lactobacillus reuteri DSM 17938 feeding of healthy newborn mice has recently been reported to positively regulate immune responses in terms of increased Foxp3^+^ Treg cells levels, as well as increase the bacterial diversity of the gut microbiota [164]. A recent animal model study evaluated the effects of streptococcus faecalis, clostridium butyricum and bacillus mesentericus on the growth and immune status of piglets [121]. Upon treatment, a rise in CD4+ and IgM+ cells isolated from the liver was described alongside a decrease of CD4+CD8+ T cell number in Peyer’s patches of treated piglets. Phagocytic activity of MHC class II+ cells isolated from the liver has also been reported to have increased [121].

The immunomodulatory effect of probiotics by mediating the cytokine release has repeatedly been demonstrated in vivo. Two probiotic strains isolated from Tibetan yaks bacillus subtilis and bacillus velezensis have been reported to improve the blood parameters [218] linked with immunity and inflammation in treated mice [122]. Moreover, an increase in IgA, IgG and IgM release has been observed in probiotic-fed mice, while at the same time the up-regulation of IL-10 and down-regulation of TNF-α, IL-6, and IL-8 was observed upon administration [122]. A significant reduction in pro-inflammatory cytokine production, including TNF-α and INF-γ by spleen cells and Peyer’s patch lymphocytes, in probiotic-treated mice has been reported [73]. An early study conducted on IL-10-deficient colitis-affected mice reported that lactobacillus salivarius and bifdobacterium infantis were able to significantly attenuate colitis, while simultaneously increasing the production of Th1-type cytokines systemically and mucosally [137]. It should be emphasized that murine colitis models have frequently been employed in order to analyze probiotics–host interactions [219,220,221]. Data obtained in recently developed mouse models demonstrated that lactobacillus casei CRL 431 can exert an anti-inflammatory response [222], while the interaction of this probiotic with gut-associated immune cells can stimulate the expression of macrophage mannose receptor CD206 and TLR-2 [223]. Further data indicated that the administration of lactobacillus acidophilus NCFB 1748 and lactobacillus paracasei subsp. paracasei in DC412 BALB/c inbred mice and Fisher-344 inbred rats favored increased chemotaxis of polymorphonuclear cells in association with increased phagocytosis and TNF-α production [143]. In particular, both probiotic strains were capable of interacting with the GALT of assayed animals by stimulating the activation of TLR2/TLR4-mediated signaling, ultimately leading to the secretion of IL-6, IL-10, IFN-γ and TNF-α. Similarly, an additional study conducted with BALB/c mice demonstrated that lactobacillus casei can induce the activation of the intestinal mucosal immune system through innate immunity mechanisms [147]. Specifically, upon lactobacillus casei administration, the expression of CD206 and TLR-2 receptors has been reported as increased in mononuclear cells from Peyer’s patches isolated from mice. Despite the lack of T cell and IL-5-positive cell number increase, an increase IgA+ and IL-6-producing cells was reported following probiotic administration. Thus, lactobacillus casei can prompt the innate immune response in vivo by increasing the expression of specific markers of immune cells without perturbing the T cell number [147]. With the same BALB/c mice model, an increase in levels of β-Defensin-1, secretory IgAs and a reduced number of staphylococcus aureus colonies in vaginal mucosa has recently been observed upon administration of lactobacillus reuteri [162]. Recently, lactobacillus casei ATCC 393 has been reported to induce the overexpression of nucleotide binding oligomeric domain-like receptor protein 3 (NLRP3), cysteine proteinase-1 (Caspase-1), IL-1β, and IL-18 in a mouse model of ulcerative colitis [149]. In particular, NLRP3 is an inflammasome that detects a broad range of microbial targets [49]. A mixture of lactobacillus paracasei and reuteri has been reported to reduce the amount of mucosal pro-inflammatory cytokines leading to an attenuation in the colitis of IL-10-deficient mice infected with helicobacter hepaticus [156]. The theorized underling mechanism provides the possible inhibitory activity of Lactobacilli on NF-kB activation in the intestinal mucosa, leading to a diminished expression of IL-12. At the same time, the absence of IL-10 in the intestine of IL-10-deficient mice might have been resulted in the lack of effect on IFN-γ release. T- and NK cells derived by IFN-γ activity might be directly suppressed by IL-10, independently of TNF-α, IL-12, or IL-18 [156]. Moreover, the downregulation of pro-inflammatory cytokines TNF-α and IL-6 and anti-inflammatory cytokine IL-10 accompanied by significant increases in IgAs/IgGs has been reported in a rat model treated with lactobacillus and bifdobacterium [224]. A reduction in IL-10 levels has also been observed in the ileum of mice treated with bifidobacterium bifidum MIMBb23sg [134]. This effect was in parallel with the downregulation of the cyclooxygenase COX-2 in the colon, thus suggesting an anti-inflammatory/regulatory activity of MIMBb23sg. Notably, increased serum IL-1β, IL-6, and TNF-α levels have been described in rats administered with lactobacillus casei, lactobacillus acidophilus, lactobacillus rhamnosus, lactobacillus bulgaricus, bifidobacterium infantis, bifidobacterium breve, and streptococcus thermophilus upon exposure to acrylamide [131]. In particular, rats exposed to acrylamide developed an increased systemic inflammation which was attenuated following probiotic administration. The effect of milk implementation with probiotics has been evaluated in animal models. Milk implemented with lactobacillus casei DN 114001 has been reported to favor the release of IL-6 as well as increase the number of different immune cell populations including macrophages, IgA+ B lymphocytes and cells from the nonspecific barrier, i.e., goblet cells. Notably, lactobacillus casei was also capable of activating the enzyme calcineurine; this activation, in turn, led to the activation of the nuclear factor of activated T cells (NFAT), which is known to positively influence several immune processes, including T-cell development, anergy, apoptosis and immune system aging [225]. A similar effect has also been reported in mice administered with lactobacillus acidophilus (strains CRL 1462 and A9), lactobacillus casei CRL 431 and escherichia coli (strains 129 and 13-7). In particular, lactobacillus acidophilus (strains CRL 1462 and A9) and lactobacillus casei CRL 431 increased the expression of TLR-9 in mice, while an increase in calcineurin expressing cell number in lamina propria has been reported upon administrating all assayed probiotics [140].

A recent study evaluated the immunomodulatory activity of lactiplantibacillus plantarum CJLP243, CJW55-10, and CJLP475 in immunodeficient mice [141]. Isolated marrow-derived macrophages from mice have been reported to release IL-6, IL-12 and INF-α in parallel with the release of co-stimulatory molecules such as CD80 and CD86. The NK cell cytotoxicity and proliferation increase was also reported [141]. A mixture of five probiotic strains, named IRT5, that includes bifidobacterium bifidum, lactobacillus acidophilus, casei, and reuteri, and streptococcus thermophilus, presented immunomodulatory activities in a mouse model of autoimmune dry eye [170]. In particular, a decrease in proteins related to antigen-presenting processes in the CD11b^+^ and CD11c^+^ cells of spleen in the IRT5-treated groups was found [170].

Animal model-based studies on probiotic effects in several diseases reported promising results [226,227,228]. The administration of a formulation of four distinct probiotic strains, i.e., lactobacillus acidophilus, lactobacillus casei, lactobacillus reuteri, streptococcus thermophilus and bifidobacterium bifidum, can contribute to suppressing immune disorders such as IBD, atopic dermatitis, and rheumatoid arthritis. The mechanisms relying on this immunomodulatory effect mediated by probiotics relies on the increase in CD4+ Foxp3^+^ regulatory Treg cells and decrease numbers of Th1, Th2, and Th17 cytokines. In particular, the conversion of T cells into Foxp3^+^ Treg cells has been reported to be directly mediated by regulatory DCs that express high levels of IL-10, TGF-beta, COX-2, and indoleamine 2,3-dioxygenase [168]. A recent comprehensive review conducted in more than 30 animal-based studies, including 28 probiotic therapy and 9 commensal therapy studies, underlined the therapeutic efficacy of several multispecies probiotic formulations. In particular, VSL#3, which is a formulation of three different bifidobacteria, four lactobacilli and one streptococcus thermophilus strain [229], as well as lactobacillus paracasei, bifidobacterium animalis, escherichia coli Nissle 1917, and even prevotella histicola, emerged as the most promising in the treatment of multiple sclerosis [126]. VSL#3 has also been reported to attenuate sickness behavior development in mice with liver inflammation without affecting disease severity, the gut microbiota composition, or gut permeability [171]. This effect was accompanied by reductions in microglial activation and cerebral monocyte infiltration as well as a decrease in TNF-α levels [171]. Furthermore, both lactobacillus and bifidobacterium have been reported to be effective in reducing anxiety-like behaviors in mice and rats [230]. Lastly, a study aimed at determining the impact of probiotic bacteria on degenerative alterations of the gut microbiota and cognitive behavior demonstrated that the administration of lactobacillus fermentum JDFM216 can increase mouse behavior, improve phagocytic activity of macrophages, enhance sIgA production, and stimulate immune cells activity [153].

Probiotics have proven to present antitumor properties in vivo [231]. The antitumor potential of Lactobacillus casei BL23 strain has been investigated in a study conducted with an HPV-induced cancer mouse allograph model [144]. In tumor-bearing BL23-fed mice, a negative correlation between local Foxp3 levels and tumor size and T-cells subpopulations has been described. Moreover, probiotic administration has been related with a local recruitment of NK cells and cytotoxic activity. These data underline the anti-tumoral potential of BL23 [144].

The probiotic efficacy in allergic diseases have been reported. Probiotics have been shown to be effective in reducing the levels of IgE, which is one of the most important players in the allergic responses. Several in vivo studies demonstrated the beneficial effect of probiotics in counteracting/preventing allergic diseases [62,232,233]. A probiotic fermented milk mixture containing four different probiotic strains, namely lactobacillus bulgaricus, streptococcus thermophilus and lactobacillus paracasei CNCMI-1518, was administered to sensitized mice. The mixture shifted the Th2 cell profile response towards a Th1 response that led to the production of IgGs instead of IgEs. At the same time, an increase in IL-10 and IFN-γ levels has been described. IFN-γ release was afterwards attributed to Th1 cells [167]. Mice treated with a mixture of lactobacillus rhamnosus and lactis presented an increase in TGF-β levels ad Treg response, thereby leading to a decrease in IgE levels and therefore the inhibition of allergic responses [161]. In an ovalbumin (OVA)-induced mouse model with allergic airway disease (AAD), which is a human asthma model, six probiotic strains, i.e., bifidobacterium breve M-16V, infantis NumRes251, animalis NumRes252 and NumRes253, lactobacillus plantarum NumRes8 and rhamnosus NumRes6, have been reported to (i) improve lung function (ii) raise the eosinophils number and (iii) increase the levels of IgE, IL-4, IL-5 and IL-10 in the bronchoalveolar lavage fluid (BALF) [127]. Furthermore, when simultaneously administered, bifidobacterium animalis and brave and lactobacillus helveticus and paracasei can improve allergic responses, thus alleviating the clinical symptoms of allergic disease [169]. Lastly, a protective effect against allergy has been reported in an early murine allergy model-based study where mice were fed with bifidobacterium breve AH1205 and bifidobacterium longum AH1206. The potential immunoregulatory activity of these probiotic strains has been demonstrated by the observation that the number of Foxp3(+) Treg cells increased upon probiotic administration [133].

In summary, studies conducted in vivo with animal models underline the immunomodulatory effect of different probiotic bacteria in managing diseases in humans.

## 7. Guidelines on the Use of Probiotics in Clinical Practice and Probiotic-Based Clinical Trials

Probiotics are described as generally recognized as safe (GRAS) by the American Food and Drug Administration (FDA) or as qualified presumption of safety (QPS) by the European Food Safety Authority (EFSA) [234,235]. However, the marketing of probiotic products is typically geared directly to consumers with no indication of their actual clinical effectiveness as a consequence of the lack of regulation of probiotic industry. This trend, which undoubtedly leads to the generation of biased information among consumers, underlines the urgency of providing appropriate indications of probiotic use by scientists/clinicians. Various national/international scientific societies/agencies, such as the American Gastroenterological Association (AGA), the European Society for Pediatric Gastroenterology, Hepatology, and Nutrition (ESPGHAN) and the European Society of Pediatric Infectious Diseases (ESPID) evaluated the clinical reliability of probiotics and formulated official recommendations for the use of these microorganisms in managing human intestinal disorders, such as diarrhea, colitis, pouchitis, IBS, Necrotising enterocolitis (NEC) and acute gastroenteritis [236,237,238,239,240]. Both pre-clinical and clinical probiotic-based studies have been evaluated. The studies were extremely varied, with profound differences in experimental design and research methodology as well as in the probiotic strain tested, dose and route of administration. Either positive or negative recommendations have been released, while, in numerous cases, insufficient data were available to make a recommendation.

The governing board of the American agency AGA selected members of the guideline panel and technical review panel to carefully evaluate the published data, taking into account the U.S. Institute of Medicine recommendations for clinical guideline development [239,240]. The quality of revised works was classified as high, moderate, low and very low, according to the level of robustness of the reported data. Overall, the quality of data was remarkably low, and the indication of potential harms was more than frequently inconsistent. However, the summary of recommendations indicates that using distinct probiotics, such as saccharomyces boulardii, or probiotic combinations such as the (i) 2-strain combination of lactobacillus acidophilus CL1285 and lactobacillus casei LBC80R, (ii) 3-strain combination of lactobacillus acidophilus, lactobacillus delbrueckii subsp. bulgaricus, and bifidobacterium bifidum, and (iii) 4-strain combination of lactobacillus acidophilus, lactobacillus delbrueckii subsp. bulgaricus, bifidobacterium bifidum, and streptococcus salivarius subsp. thermophilus could support the prevention of clostridioides difficile infection, which causes diarrhea and colitis, for adults/children under antibiotic therapy [239,240]. In adults and children with pouchitis, the AGA committee reported the efficacy of the 8-strain combination of lactobacillus paracasei subsp. paracasei, lactobacillus plantarum, lactobacillus acidophilus, lactobacillus delbrueckii subsp. bulgaricus, bifidobacterium longum subsp. longum, bifidobacterium breve, bifidobacterium longum subsp. infantis, and Streptococcus salivarius subsp. thermophilus. For the prevention of NEC in preterm (less than 37 weeks gestational age), low-birth-weight infants, the AGA committee recommended various probiotic combinations such as (i) lactobacillus subsp. and bifidobacterium subsp. (lactobacillus rhamnosus ATCC 53103 and bifidobacterium longum subsp. infantis, (ii) lactobacillus casei and bifidobacterium breve; lactobacillus rhamnosus, lactobacillus acidophilus, lactobacillus casei, bifidobacterium longum subsp. infantis, bifidobacterium bifidum, and bifidobacterium longum subsp. longum, (iii) lactobacillus acidophilus and bifidobacterium longum subsp. infantis; (iv) lactobacillus acidophilus and bifidobacterium bifidum, (v) lactobacillus rhamnosus ATCC 53103 and bifidobacterium longum Reuter ATCC BAA-999, (vi) lactobacillus acidophilus, bifidobacterium bifidum, bifidobacterium animalis subsp. lactis, and bifidobacterium longum subsp. longum), (vii) bifidobacterium animalis subsp. lactis (including DSM 15954), (viii) lactobacillus reuteri (DSM 17938 or ATCC 55730), and (ix) lactobacillus rhamnosus (ATCC 53103 or ATC A07FA or LCR 35). Lastly, given the significant knowledge gap about Crohn’s disease, IBS, and ulcerative colitis being reported, the use of probiotics as therapeutics for the clinical management of these diseases has been discouraged.

The European scientific associations ESPGHAN and ESPID released a medical position paper and clinical guidelines for the management of acute gastroenteritis with probiotics in children for practitioners at all healthcare levels, such as pediatricians and physicians [236,238]. Given the pre-clinical and clinical data available, the committee focused its attention on six taxonomic groups, namely lactobacillus, bifidobacterium, saccharomyces, streptococcus, enterococcus, and bacillus. Based on the consistent amount of evidence in various pre-clinical and clinical settings in children with acute gastroenteritis, active treatment with probiotics such as lactobacillus rhamnosus GG and saccharomyces boulardii, in addition to rehydration therapy, has been reported to be strongly effective in reducing disease duration and symptoms [236,238]. Lactobacillus reuteri DSM 17938 and heat-inactivated lactobacillus acidophilus LB have also been included in the list of recommended strains for acute gastroenteritis management. In contrast, the use of enterococcus faecium (SF68 strain) has been discouraged because of safety issues [236,238]. Because of the insufficient/low-quality evidence available, numerous probiotic strains, largely belonging to the lactobacillus group, have not been recommended [236,238].

Although numerous studies have been published in recent years, the clinical evidence of probiotic efficacy in managing human intestinal disorders is either weak or preliminary. The significant knowledge gaps alongside the lack of clinical application of preclinical data lead to the recommendation of further high-quality studies that may address these issues.

In recent years, a significant number of investigators and/or clinicians have developed an increasing number of randomized clinical trial protocols for evaluating the probiotic efficacy in improving human health/immune function and counteracting/preventing diseases [234,241,242]. On 3 October 2022, by using the search term “probiotics”, a total of 1487 trials, focused more on children rather than on the elderly >65 years of age, were included in the online database ClinicalTrials.gov (www.clinicaltrial.gov, accessed on 3 October 2022). Among these, 304 trials are currently ongoing. Although various diseases/conditions are addressed, the most frequently studied conditions include gastrointestinal diseases. To a lesser extent, non-gastrointestinal conditions, such as infection-related, communicable, and metabolic diseases, have also been registered. Additional clinically investigated diseases/conditions include allergic, cardiovascular, and neurodegenerative diseases. Lactobacillus, bifidobacterium, and streptococcus represent the most frequently reported probiotic genera in ClinicalTrials.gov, while lactobacillus rhamnosus GG is the most frequently studied probiotic strain, followed by bifidobacterium animalis subsp. lactis BB1 [234]. The clinical application of probiotics has been demonstrated in randomized clinical trials conducted with (i) lactobacillus casei, bifidum, fermentum and acidophilus for the treatment of Alzheimer’s disease, (ii) lactobacillus GG for cystic fibrosis, (iii) lactobacillus plantarum WCFS1 for managing autism spectrum disorders, and (iv) bifidobacteria infantis NLS, longum CECT 7347 and breve BR03/B632 for the treatment of celiac disease. Potential applications of probiotic bacteria include the prevention of urinary tract infections with lactobacillus GG, and radiation-related symptoms with lactobacillus casei DN-114001. Notably, the clinical importance of probiotics such as lactobacillus reuteri has also been underlined in clinical trials focused on cancer therapy with immune checkpoint inhibitors (ICIs) [243,244], as well as in counteracting side-effects, e.g., colitis, which might occur during ICI-based therapies [245]. Regarding the multispecies probiotic formulations, a limited number of clinical trials reported the precise description of the formulation [234]. However, VSL#3 [229] is the most frequently registered formulation in ClinicalTrials.gov. In addition to probiotic-based trials, several clinical trials focused on investigating the human microbiota were registered. The majority is focused on identifying novel commensal bacterial species with probiotic characteristics in relation to a specific clinical condition and/or disease. Consistently, approximately one quarter of registered microbiota-based clinical trials are observational studies [229].

Various clinical trials have been developed for evaluating the probiotic efficacy in improving the immune function [8]. A randomized human clinical trial indicated that probiotic formulations such as bifidobacterium infantis R0033, bifidobacterium bifidum R0071, and lactobacillus helveticus R005 can enhance the mucosal immunity of healthy infants; in particular, high levels of fecal sIgAs were reported [178]. Another randomized clinical trial conducted on healthy adult subjects which consumed low-fat milk containing bifidobacterium lactis HN019 reported an immune function boost in terms of polymorphonuclear and NK cell activity increase [246]. Similarly, milk supplemented with bifidobacterium lactis HN019 has been shown to increase the total helper (CD4+) and activated (CD25+) T and NK cells in healthy elderly volunteers. However, this immunomodulatory effect occurred in parallel with the lack of alteration in the proportions of CD8+ (MHC I-restricted T cells), CD19+ (B lymphocytes), and human leukocyte antigen including HLA-DR+ (MHC II-bearing antigen-presenting cells) [8]. The results of a double-blinded, placebo-controlled, randomized, factorial cross-over clinical trial conducted on healthy adults indicated that bifidobacterium animalis combined with xylo-oligosaccharide could induce bifidogenesis as well as modulate markers of the immune function in healthy adults, particularly reducing the expression of CD19 on B cells [247]. Interestingly, probiotic supplementation during the gestation period can potentially influence the immune parameters as well as immunomodulatory factors in breast milk. Both lactobacillus rhamnosus HN001 and bifidobacterium lactis HN019 administered to pregnant females demonstrated the ability to increase the cord blood levels of IFN-γ, while increased IgA and TGF-β1 levels were also observed in early breast milk samples [248]. Lastly, bifidobacterium infantis 35624 has been reported to decrease the proportion of C-reactive protein and pro-inflammatory cytokines in patients suffering from ulcerative colitis and chronic fatigue as well as increase the levels of Foxp3^+^ Treg lymphocytes in the peripheral blood of healthy volunteers [249,250]. These findings, obtained from clinical trials, demonstrate that probiotics can positively modulate the humoral immune response function in humans.

Regarding the non-specific cellular immune response, several clinical trials demonstrated that consumed probiotics could stimulate phagocytic activity in humans. An early clinical trial, based on the consumption of milk supplemented with lactobacillus acidophilus strain La1 and bifidobacterium bifidum by healthy adult individuals, demonstrated that phagocytic activity of blood leukocytes, particularly granulocytes, can increase upon probiotic consumption [251]. Similarly, the consumption of milk containing bifidobacterium lactis HN019 by a group of healthy elderly subjects has been reported to increase the polymorphonuclear cell phagocytic capacity [135]. Another randomized clinical trial conducted on healthy volunteers reported similar conclusions, indicating that consumed yogurt supplemented with lactobacillus acidophilus 74-2 and bifidobacterium lactis 420 can favor the increase in the overall phagocytic activity of granulocytes and monocytes [252]. These findings cumulatively underline the pivotal role of probiotics in immune function regulation in humans, through the activation of important immune signaling pathways that modulate the activity of immune cells. The underlying mechanism of action of probiotic bacteria in improving the immune functions needs to be further investigated.

In summary, based on the clinical data currently available, probiotics present beneficial and multifaceted effects on human health, which encourage further clinical research. Novel clinical trials should be performed in order to confirm the beneficial effect of probiotics in managing specific diseases, by understanding the specific dose, therapy duration and possible side effects, as well as to identify novel clinically reliable probiotic strains.

## 8. Probiotic Industrial Production Challenges

A huge number of preclinical studies have been conducted, while others, aimed at characterizing and isolating novel potential probiotic bacteria candidates, are still ongoing; clinical trials are also currently in continuous development. However, at the same time, little information has yet obtained on probiotic-based industrial processes. In general, numerous probiotic strains fail to reach commercialization, or the information behind probiotic preparation and industrial production is under restricted access, as industrial secrets, and/or under patent [253]. The majority of probiotic strains belong to the list of microorganisms that are safe for human consumption, i.e., GRAS or QPS. However, ensuring the safety of probiotics is a fundamental step that should be taken to industrialize the product. The selected strains are deposited into freely accessible collections and their genetic identity is continually ensured. In case of the safety and efficacy of probiotics being determined, emphasis is placed on the design and optimization of their industrial production and applicability. Commercial products are expected to have specific features such as high cell viability and stable cell concentration with consistent behavior, depending on the field for which they are typically designed [254]. A weakness in this context is that laboratory-produced bacteria may not perform in terms of physiology and viability when growing on an industrial scale. In other words, probiotics may not maintain their properties. Pilot-scale tests should therefore be performed to evaluate the effect of the process operations on microorganisms’ characteristics, while their viability must also be determined.

The probiotic industrial process follows a number of different stages. The process begins with the inoculum of probiotic bacteria into the fermenter, where they later undergo sequential fermentation phases until the desired volume of biological material is reached. The main goal of the probiotic industrial process is to limit the number of generations between the inoculum and the final product in order to minimize any risk of genetic variation. Several problems might arise in the industrial production of probiotics. Among these, of particular importance is the problem of probiotic stability, which is still unsolved. Probiotic mass production in a bioreactor requires the maintenance of strict conditions to allow for the most efficient microorganism viability and growth. The most important conditions comprise various medium formulations, optimal temperatures and pH, as well as adequate H_2_O and oxygen levels inside the bioreactor. All these conditions can vary greatly according to the type of probiotic strain being considered [255,256]. The product manufacturing and storage processes may affect the viability of bacterial strains, thus influencing the stability and healthy properties of probiotics. The freezing and/or lyophilization processes, which can potentially damage probiotic cells and reduce their viability, can be prevented by using cryoprotectants and lyoprotectants [257]. Dried cell rehydration is also essential to maximize the productivity of the probiotics [258], and therefore plays a critical role in the biomass production at a commercial level. Optimal culture medium and cell protectants selection is thus essential to increase the efficacy of the probiotic product. Most probiotic strains are either strictly anaerobic or facultatively anaerobic. Thus, in order to reduce redox potential, oxygen permeation in vectors should be reduced or, alternatively, oxygen scavengers should be introduced [259]. The viability of probiotics after consumption is another important point to be considered. Indeed, the bacterial strains should remain viable in adequate numbers during the passage throughout the gastrointestinal tract. Probiotic bacteria protection can be improved by microencapsulation, which improves the adaptation of the probiotic to the gastrointestinal tract conditions, improving the stability of the strain [260]. Currently, fermented milk and yogurts are the best-known probiotic carriers on the market. Nevertheless, certain probiotic strains are sensitive to oxygen and pH in fermented products. In turn, this sensitivity can affect the stability of probiotics by post-acidification, while being stored in the fridge. To minimize this phenomenon, it is necessary to select strains without post-acidify potential [261]. This may represent an economic burden on manufacturers and limit the addition of probiotics to various products [262]. The reproducibility of the probiotic product is an additional critical aspect of their industrial production. Several attempts have been made to determine the number of viable probiotic strains in the products, but without success [259]. Another challenge in the production of biomass from probiotic cells is represented by the (i) conditions that can affect the functional properties of probiotic cells [258], and (ii) timing of probiotic harvesting [263]. Lastly, the challenge in biomass production of probiotic cells is also the economic aspect, which is the backbone of any industrial or commercial production [264]. Inexpensive production will correspond to high sales and therefore a high number of consumers. The cost in the market makes it easy for users or consumers to buy probiotics for their consumption.

Probiotic industrial production provides several legislative issues. Probiotics are classified under different categories depending on the country being considered, while their name and use as functional foods can also vary. For instance, given that probiotics are currently not legally defined in Europe, they are included on the QPS provided by EFSA and are indicated as functional foods. The QPS list is updated periodically to reflect the safety assessment of probiotics recommended for inclusion, but not all probiotics are eligible for approval [265,266]. Similarly, in the U.S.A., GRAS products should be approved by the FDA. Whether a specific probiotic is used as a dietary supplement it can be considered as food, and it is therefore under the Dietary Supplement Health and Education Act (DSHEA) regulation. On the other hand, if a probiotic is designed for therapeutic purposes, the probiotic medicinal product should be verified as safe and efficacious by FDA. However, as far as both FDA and EFSA are concerned, probiotics cannot be used in health claims. In Japan, efficacy claims of probiotic products are forbidden on the labeling until permission from the Ministry of Health and Welfare (MHLW) has been granted that allows the product to be considered as Foods for Specific Health Uses (FOSHU); the probiotic should present a mandatory validation of efficacy and safety [267]. Considering that the definition and classification of probiotics by regulators is different worldwide, the status of probiotics as commercial products remains unclear. Thus, regulators, producers and consumers may have concerns about probiotic product claims.

## 9. Concluding Remarks

Broad evidence indicates that intestinal immune cells interact with consumed probiotics, and this interaction can improve host immune homeostasis and immune function [268]. Although probiotics have been studied for a long time, a relatively restricted number of studies have described the molecular mechanisms underlying the immunomodulatory functions of probiotic bacteria and how they are able to interact with host immune cells [269,270,271]. Consumed probiotics specifically mediate the activation/modulation of both innate and adaptive immune responses in the intestine by stimulating the (i) production of various cytokines and chemokines from DCs, lymphocytes, macrophages, mast cells, granulocytes, and intestinal epithelial cells, and (ii) IgA-producing cells and consequent IgA secretion [272,273,274,275]. Probiotics can therefore improve the host immune system and induce important beneficial effects, allowing the prevention and/or management of immune/inflammatory-related diseases [276], including IBD [277,278], IBS [279], inflammation [280], diarrhea [281], pathogenic infections [282], infant colic, and certain cancer types [283,284,285,286]. Although improvements have been made in the field, the mechanisms of interaction between consumed probiotics and intestinal immune cells have not been well described [12,287,288]. In this context, further pre-clinical and clinical research should be performed to clarify the underlying mechanisms [289,290]. Novel precise mechanistic data should be collected in order to better understand the relationship between immune cells and probiotics and the well-established probiotic-mediated improvement of the immune system.

## Figures and Tables

**Table 1:** Beneficial effects of probiotics reported in pre-clinical studies.

Probiotic Strains	Associated Health Benefits	Experimental Model	References
Bacillus			
Bacillus mesentericus	Immunestimulation	in vivo	[121]
Bacillus subtilis	Attenuates inflammation, Immunestimulation	in vivo	[122]
Bacillus velezensis	Attenuates inflammation, Immunestimulation	in vivo	[122]
Bifidobacterium			
Bifidobacterium	Immunestimulation	in vitro	[123]
Bifidobacterium animalis DN-173 010	Immunestimulation	Elderly subjects	[124]
Bifidobacterium animalis	Immunestimulation (salivary cytokine release)	Healthy adults	[125]
Bifidobacterium animalis	Multiple sclerosis therapy	in vivo	[126]
Bifidobacterium animalis NumRes252/-253	Improve lung function, Immunestimulation	in vivo	[127]
Bifidobacterium animalis subsp. Lactis	Imunnemodulation	in vitro	[98,128,129]
Bifidobacterium bifidum	Immunestimulation (cytokine release)	in vitro	[130]
Bifidobacterium breve	Immunestimulation (cytokine release)	in vivo	[131]
Bifidobacterium breve IPLA 20004	Improves intestinal barrier function	in vitro	[132]
Bifidobacterium breve M-16V	Improves lung function, Immunestimulation	in vivo	[127]
Bifidobacterium breve AH1205	Immunestimulation	in vivo	[133]
Bifidobacterium breve UCC2003	Imunnemodulation	in vitro, in vivo	[98,128,129]
Bifidobacterium bifidum MIMBb23sg	Attenuates inflammation	in vivo	[134]
Bifidobacterium bifidum LMG13195	Improves intestinal barrier function	in vitro	[132]
Bifidobacterium lactis HN019	Immunestimulation	Elderly subjects	[135]
Bifidobacterium longum	Immunestimulation (cytokine release)	in vitro	[130]
Bifidobacterium longum	Imunnemodulation	in vitro	[136]
Bifidobacterium longum AH1206	Immunestimulation	in vivo	[133]
Bifidobacterium infantis NumRes251	Improves lung function, Immunestimulation	in vivo	[127]
Bifdobacterium infantis	Attenuates colitis, Immunestimulation (cytokine release)	in vivo, in vivo	[131,137]
Clostridium butyricum	Immunestimulation	in vivo	[121]
Escherichia coli	Immunestimulation to inactivated influenza vaccine	in vivo	[138]
Escherichia coli	Immunestimulation (cytokine release)	in vitro	[139]
Escherichia coli 129	Imunnemodulation	in vivo	[140]
Escherichia coli 13-7	Imunnemodulation	in vivo	[140]
Escherichia coli Nissle 1917	Multiple sclerosis therapy	in vivo	[126]
Lactiplantibacillus plantarum CJLP243/-45/-W55-10	Immunestimulation (cytokine release)	in vivo	[141]
Lactobacillus			
Lactobacillus acidophilus	Immunestimulation (cytokine release)	in vitro, in vivo	[130,131]
Lactobacillus acidophilus	Immunestimulation (IgA-producing cells increase)	Healthy adults	[119]
Lactobacillus acidophilus CRL 1462	Imunnemodulation	in vivo	[140]
Lactobacillus acidophilus A9	Imunnemodulation	in vivo	[140]
Lactobacillus acidophilus NCFM	Immunestimulation (cytokine release)	in vitro	[142]
Lactobacillus acidophilus NCFB 1748	Increased chemotaxis of polymorphonuclear cells	in vivo	[143]
Lactobacillus bulgaricus	Immunestimulation (cytokine release)	in vitro, in vivo	[131,139]
Lactobacillus casei BL23	Antitumor proprieties	in vivo	[144]
Lactobacillus casei CRL 431	Imunnemodulation	in vivo	[140]
Lactobacillus casei CRL 431	Immunestimulation	in vitro	[120,145,146]
Lactobacillus casei	Immunestimulation (cytokine release)	in vitro, in vivo	[130,131,139,147]
Lactobacillus casei	Reduces symptoms of rotavirus diarrhea	Children with rotavirus diarrhea	[148]
Lactobacillus casei	Immunestimulation (IgA-producing cells increase)	Healthy adults	[119]
Lactobacillus casei ATCC 393	Attenuates colitis, Immunestimulation	in vivo	[149]
Lactobacillus casei Shirota	Immunestimulation (salivary cytokine release)	Healthy adults	[125]
Lactobacillus casei IMAU60214	Immunestimulation (cytokine release)	in vitro	[150]
Lactobacillus crispatus	Immunestimulation (cytokine release)	in vitro	[139]
Lactobacillus delbrueckii subsp. bulgaricus	Immunestimulation (IgA-producing cells increase)	Healthy adults	[119]
Lactobacillus johnsonii	Immunestimulation (cytokine release)	in vitro	[151]
Lactobacillus johnsonii NBRC 13952	Immunestimulation (cytokine release)	in vitro	[152]
Lactobacillus fermentum	Immunestimulation (salivary cytokine release)	Healthy adults	[125]
Lactobacillus fermentum JDFM216	Increases mouse behavior, Immunestimulation	in vivo	[153]
Lactobacillus gasseri SBT2055	Immunestimulation (IgA-producing cells increase)	in vivo	[154]
Lactobacillus helveticus R389	Immunestimulation	in vitro	[120]
Lactobacillus helveticus IMAU70129	Immunestimulation (cytokine release)	in vitro	[150]
Lactobacillus lactis	Immunestimulation (IgA-producing cells increase)	Healthy adults	[119]
Lactobacillus salivarius	Attenuates colitis	in vivo	[137]
Lactobacillus paracasei	Multiple sclerosis therapy	in vivo	[126]
Lactobacillus paracasei	Immunestimulation	in vitro, in vivo	[155]
Lactobacillus paracasei CNCM I-1518	Immunestimulation	in vitro, in vivo	[145]
Lactobacillus paracasei + reuteri	Attenuates inflammation and colitis	in vivo	[156]
Lactobacillus paracasei subsp. Paracasei	Increases chemotaxis of polymorphonuclear cells	in vivo	[143]
Lactobacillus plantarum	Immunestimulation (IgA-producing cells increase)	Healthy adults	[119]
Lactobacillus plantarum	Immunestimulation (CD40 and CD80 expression increase)in vitro	[157]	
Lactobacillus plantarum	Immunestimulation	in vitro, in vivo	[98,158]
Lactobacillus plantarum N14	Immunestimulation	in vitro	[159]
Lactobacillus plantarum NumRes8	Improve lung function, Immunestimulation	in vivo	[127]
Lactobacillus rhamnosus	Immunestimulation (cytokine release)	in vitro, in vivo	[130,131]
Lactobacillus rhamnosus	Immunestimulation (IgA-producing cells increase)	Healthy adults	[119]
Lactobacillus rhamnosus GR-1	Immunestimulation	HIV/AIDS-affected patient	[160]
Lactobacillus rhamnosus GG	Reduces symptoms of rotavirus diarrhea	Children with rotavirus diarrhea	[148]
Lactobacillus rhamnosus GG	Immunestimulation (cytokine release)	in vitro	[150]
Lactobacillus rhamnosus KLSD	Immunestimulation (cytokine release)	in vitro	[150]
Lactobacillus rhamnosus NumRes6	Improves lung function, Immunestimulation	in vivo	[127]
Lactobacillus rhamnosus	Immunestimulation (salivary cytokine release)	Healthy adults	[125]
Lactobacillus rhamnosus + lactis	Attenuates allergic disease	in vivo	[161]
Lactobacillus reuteri	Immunestimulation	in vitro	[162]
Lactobacillus reuteri RC-14	Immunestimulation	HIV/AIDS-affected patient	[160]
Lactobacillus reuteri ATCC 55730	Reduces symptoms of rotavirus diarrhea	Children with rotavirus diarrhea	[163]
Lactobacillus reuteri DSM 17938	Gut microbiota diversity increase, Immunestimulation	in vivo	[164]
Lactobacillus reuteri 100-23	Immunestimulation (cytokine release)	in vitro	[165]
Lactobacillus sakei	Immunestimulation (cytokine release)	in vitro	[151]
Lactocaseibacillus			
Lacticaseibacillus paracasei SD1	Immunestimulation (cytokine release)	in vitro	[166]
Lacticaseibacillus rhamnosus SD4	Immunestimulation (cytokine release)	in vitro	[166]
Lacticaseibacillus rhamnosus SD11	Immunestimulation (cytokine release)	in vitro	[166]
Limosilactobacillus fermentum SD7	Immunestimulation (cytokine release)	in vitro	[166]
Prevotella histicola	Multiple sclerosis therapy	in vivo	[126]
Streptococcus			
Streptococcus thermophilus	Immunestimulation (cytokine release)	in vitro, in vivo	[130,131]
Streptococcus thermophilus	Immunestimulation (IgA-producing cells increase)	Healthy adults	[119]
Streptococcus faecalis	Immunestimulation	in vivo	[121]
Probiotic Mixtures			
Lactobacilli+ Streptococchi	Immunestimulation	in vivo	[167]
Lactobacilli+ Streptococchi +			
Bifidobacteria	Imunnemodulation	in vitro	[168]
Bifidobacteria + Lactobacilli	Attenuates allergic disease	in vivo	[169]
IRT5	Immunestimulation	in vivo	[170]
VSL#3	Attenuates sickness behavior development	in vivo	[171]

Abbreviations: Immunodeficiency syndrome (HIV/AIDS). Probiotic mixtures: IRT5: bifidobacterium bifidum, lactobacillus acidophilus, casei and reuteri, and streptococcus thermophiles; VSL#3: 3 different bifidobacteria, 4 lactobacilli, and 1 streptococcus thermophilus strain; bifidobacteria + Lactobacilli: bifidobacterium animalis + brave + lactobacillus helveticus + paracasei.

## Data Availability

Not applicable.
